# Effects of Third-Wave Cognitive Behavioral Therapy for Healthcare Professionals’ Burnout: A Systematic Review and Meta-Analysis

**DOI:** 10.3390/healthcare13243253

**Published:** 2025-12-11

**Authors:** Jin-Hui Han, Miran Lee, Chiyoung Cha, Gumhee Baek

**Affiliations:** 1 College of Nursing, Ewha Womans University, Seoul 03760, Republic of Korea; 2 Ewha Research Institute of Nursing Science, College of Nursing, Ewha Womans University, Seoul 03760, Republic of Korea

**Keywords:** health personnel, burnout, professional, cognitive behavioral therapy, meta-analysis, systematic review

## Abstract

**Highlights:**

**What are the main findings?**

- There has been an increase in the use of third-wave cognitive behavioral therapy (CBT) to address stress and burnout among healthcare professionals
- Emotional exhaustion and depersonalization are the most severe and prevalent burnout dimensions in healthcare settings, but third-wave CBT can significantly reduce these symptoms.

**What are the implication of the main findings?**

- Limited improvement is observed in personal accomplishment, suggesting influence of organizational factors.
- There is a need for combined psychological and organizational strategies to effectively address all burnout dimensions

**Abstract:**

**Background/Objectives**: Burnout, caused by chronic workplace stress, encompasses emotional exhaustion, depersonalization, and low personal accomplishment. Over half of healthcare professionals experience burnout, leading to increased turnover. Third-wave cognitive behavioral therapies are effective in managing burnout. This study aimed to synthesize existing evidence on third-wave cognitive behavioral therapies and evaluate its effectiveness in reducing burnout among healthcare professionals. **Methods**: We systematically searched eight databases for studies published through May 2024. The eligible studies included randomized controlled trials and quasi-experimental designs evaluating third-wave cognitive behavioral therapies on burnout in healthcare professionals. A meta-analysis was conducted by applying fixed- and random-effects models. **Results**: A total of 5005 records were identified, of which 29 were reviewed and summarized, and 11 were included in the meta-analysis. Most interventions utilized mindfulness-based techniques (*n* = 26) with delivery methods including on-site (*n* = 16), online (*n* = 12), and hybrid (*n* = 1) formats. Third-wave cognitive behavioral therapies significantly reduced emotional exhaustion (standardized mean difference [SMD] = −0.686, confidence interval [CI] = −1.237, −0.136, *p* = 0.0145, I^2^ = 92.5%) and depersonalization (SMD = −0.529, CI = −0.975, −0.083, *p* = 0.0202, I^2^ = 89.3%), but the effect on low personal accomplishment was not statistically significant (SMD = 0.311, 95% CI = −0.319 to 0.941, *p* = 0.3338, I^2^ = 89.4%). **Conclusions**: Third-wave cognitive behavioral therapies should be implemented to reduce emotional exhaustion and depersonalization; future research should target low personal accomplishment and explore approaches beyond mindfulness.

## 1. Introduction

Burnout is a syndrome resulting from prolonged exposure to chronic workplace stress that has not been effectively managed [1]. Three burnout dimensions have been identified: emotional exhaustion (EE), depersonalization (DP), and low personal accomplishment (PA) [2]. EE includes feelings of depletion and being emotionally overextended, DP refers to detached or impersonal response toward one’s clients or patients, and PA indicates perceived decreases in competence and achievement [2]. These dimensions can manifest differently with distinct antecedents and consequences [3]. Therefore, it is critical to investigate each burnout dimension separately to gain a better understanding of the phenomenon. However, these dimensions may emerge through different mechanisms and require separate consideration to fully understand the impact of burnout on healthcare professionals (HPs).

Recent meta-analyses have reported that more than half of the healthcare professionals (HPs) worldwide experience burnout, with particularly high rates of EE and DP [4]. This results in a decline in job satisfaction, an increase in workplace turnover, and a deterioration in the quality of patient care [5,6]. Burnout among HPs is significantly associated with suicidal ideation [7], decreased job satisfaction [8], and increased turnover intention, ultimately resulting in a loss of HPs and deterioration in the quality of care, which is also negatively affected, as is patient safety and health status [9,10,11]. Consequently, burnout among HPs is recognized as a global societal issue that needs to be addressed, as it affects public health. However, previous studies have often included both healthcare and non-healthcare workers, making it difficult to generalize the findings to healthcare-specific contexts. The COVID-19 pandemic also accelerated the adoption of digital interventions such as mindfulness-based and acceptance-based therapies [12,13], highlighting the need to evaluate these approaches specifically in HP-centered contexts.

A notable shift in the focus of cognitive behavior therapy (CBT) has resulted in third-wave CBT approaches, which prioritize altering psychological contexts and cognitive attitudes that precipitate psychosocial health issue development [14]. These include mindfulness-based cognitive [8], acceptance and commitment (ACT) [15], rational emotional behavior [16], and dialectical behavior [17] therapies. The initial iteration of CBT is centered on implementing behavioral modification techniques, such as classical conditioning. These techniques employ behavioral principles, including reinforcement and punishment, to alter maladaptive behaviors [18]. Second-wave CBTs emphasizes the significance of cognitive elements focusing on the identification and modification of negative thought patterns through cognitive distortion correction and transition to more realistic and productive modes of thinking to effectively resolve problems [19]. Third-wave CBTs, however, represent a shift from earlier models by emphasizing contextual processes—particularly mindfulness, acceptance, and psychological flexibility—rather than solely modifying cognitive content [14].

Recent studies have demonstrated the positive effects of third-wave CBTs on individuals experiencing burnout or depression [20,21]. The efficacy of mindfulness-based methods and ACT is well established, with significant reductions in depression and anxiety symptoms [22,23]. These findings suggest that third-wave CBTs may enhance psychological flexibility and emotion regulation-mechanisms aligned with the unique stressors faced by HPs. Despite the growing body of research on third-wave CBTs, there is a paucity of comprehensive analyses of their effectiveness, specifically for HPs experiencing burnout.

Although several meta-analyses have examined the efficacy of diverse interventions for burnout among HPs, our study is distinct in several fundamental respects. Previous systematic reviews and meta-analyses have frequently focused on specific CBTs or subsets of HPs. For example, meta-analysis studies on mindfulness-based interventions for nurses [24,25] and a systematic review of mindfulness [26] were conducted to retrieve evidence for CBT for HP burnout. However, third-wave CBTs often involve combinations of multiple techniques such as cognitive restructuring, relaxation training, mindfulness meditation, and self-compassion exercises [12,27]. Furthermore, HPs typically work in collaboration with client care, which means that they may collectively experience burnout [28]. The interconnected nature of HPs necessitates an interdisciplinary approach for addressing burnout.

Our study provides a comprehensive analysis of third-wave CBTs delivered to interdisciplinary HPs, which permits the evaluation of the distinctive contributions of several sophisticated therapeutic techniques for addressing burnout. Moreover, in contrast to previous studies that predominantly evaluated overall burnout scores, our objective was to analyze the effect sizes for each burnout dimension. This approach facilitates a more detailed examination of the impact of third-wave CBTs on burnout dimensions. Furthermore, this study integrates post-pandemic research findings to reflect the transformed healthcare delivery landscape and current modes of intervention. It aims to synthesize existing evidence on third-wave CBTs and evaluate its effectiveness in reducing burnout dimensions, EE, DP, and low PA among HPs. Accordingly, the research question was as follows: “What are the effects of third-wave CBTs on reducing burnout among healthcare professionals?”

## 2. Methods

### 2.1. Study Reporting and Registration

We conducted a systematic review and meta-analysis adhering to the Preferred Reporting Items for Systematic Reviews and Meta-Analyses (PRISMA) guidelines [29]. The protocol was registered with the International Prospective Register of Systematic Reviews (PROSPERO) under registration number CRD42024571625.

### 2.2. Eligibility Criteria

We employed the Population, Intervention, Comparison, Outcomes, and Study Design framework to structure our research question and guide the selection of relevant published literature [30]. We restricted the study designs to randomized controlled trials (RCTs) and quasi-experimental studies to ensure that the pooled effect estimates were based on interventions with adequate methodological rigor and the ability to infer causal relationships.

- Population: HPs providing care in hospitals, including physicians/surgeons, residents, interns, dentists, pharmacists, registered nurses, licensed practical/vocational nurses, and nursing assistants; no minimum requirements for years of professional experience or duration of employment were applied.
- Interventions: Third-wave CBTs (e.g., mindfulness, acceptance, and commitment therapy).
- Comparison: No intervention or alternative interventions.
- Outcome: Burnout (any validated instrument used to measure burnout was accepted for the systematic review; however, for the meta-analysis, only studies that assessed burnout using the Maslach Burnout Inventory [MBI] were included to ensure consistency and comparability of EE, DP, and PA outcomes).
- Study Design: RCTs or quasi-experimental studies.

### 2.3. Data Sources and Search Strategy

From 3 July to 12 September 2024, two researchers conducted a literature search and selected studies for this research. No publication date limits were applied, and all available articles up to August 2024 were considered. The database search was based on the Core, Standard, Ideal model presented by the National Library of Medicine; seven global search engines in English (Cochrane Library, Cumulative Index to Nursing and Allied Health Literature, Embase^®^, ProQuest Dissertations & Theses Global, PsycINFO, PubMed, and Web of Science) and one Korean search engine in Korean (Research Information Sharing Service). The literature search was limited to studies published in English or Korean. Various combinations of search terms (see Box 1) were used to either broaden or narrow the search depending on the results in a specific database. Because burnout is often comorbid with other mental health outcomes such as depression and anxiety, these terms were also included in the search strategy to ensure comprehensive coverage of relevant interventions. Boolean logic was applied consistently across all databases using the “AND” and “OR” operators, without employing controlled vocabulary. Truncation was used to broaden the search by capturing multiple forms and alternative keyword endings. In selecting the search fields, the title and abstract were selected to exclude irrelevant information such as the author, table of contents, date, and department. Throughout the review process, two researchers independently screened the records and extracted data using Covidence (https://covidence.org) [31], which automatically flagged any conflicts between reviewers. All discrepancies were jointly reviewed and resolved by all four members of the research team during weekly meetings, ensuring consistency and consensus across the entire review process. Because Covidence records only the presence of conflicts and the number of disagreements was extremely small, it was not feasible to calculate a reliable inter-reviewer agreement statistic such as Cohen’s kappa.

Box 1 **Query box.**

**Population (Healthcare professionals):** medical team; healthcare worker; healthcare provider; health personnel; doctor; physician; surgeon; nurse; midwife; licensed practical nurse; licensed vocational nurse; nurse practitioner; physician assistant; pharmacist; therapist; nurse assistant

**Intervention (Third-wave CBT):** cognitive behavioral therapy; acceptance and commitment therapy; mindfulness; behavioral activation; metacognitive therapy; dialectical behavior therapy; compassion-focused therapy

**Outcomes (Burnout):** burnout; emotional exhaustion; depersonalization; personal accomplishment

**Study design (Intervention studies):** randomized controlled trial; controlled clinical trial; intervention; quasi-experimental

### 2.4. Study Selection

**Figure 1:**
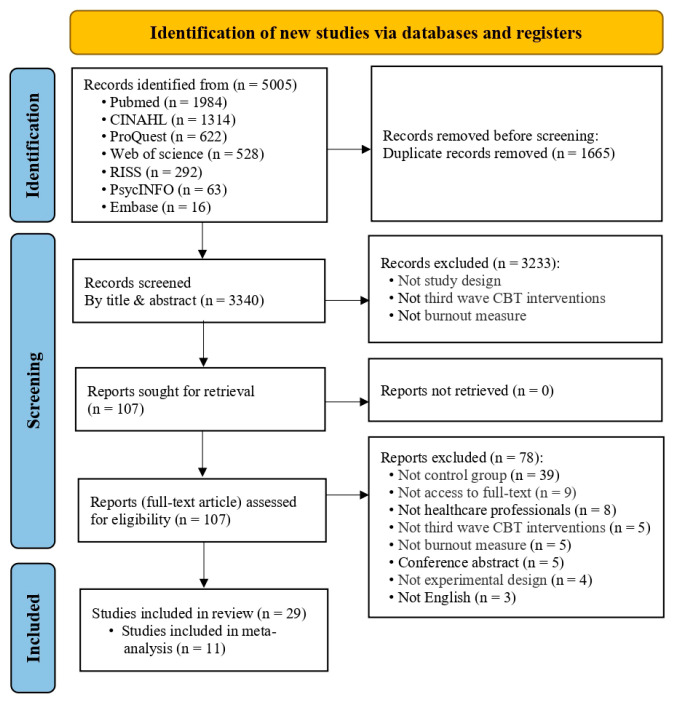
PRISMA 2020 flow diagram of study selection. Abbreviation: CINAHL = Cumulative Index to Nursing and Allied Health Literature; RISS = Research Information Sharing Service.

Literature was downloaded using the Endnote© Version X9 program with a file extension and uploaded to the literature review software Covidence for screening study titles, abstracts, and full texts [31]. The study selection process adhered to PRISMA 2020 guidelines [29], and the numerical results of each screening stage are presented in the Results section and illustrated in the PRISMA flow diagram (Figure 1).

### 2.5. Data Extraction

Data were extracted independently by four researchers who were divided into two groups. After collectively reviewing the complete texts, instances of disagreement were discussed among the researchers until a consensus was reached during weekly meetings. Data related to study characteristics included the authors, publication years, study participants, interventions, follow-up time points, and measurement tools. Data related to the intervention characteristics included the content of the intervention, mode of delivery, and dose. A standardized Google-spreadsheet–based extraction sheet was created to consistently record both study and intervention characteristics.

### 2.6. Assessment of Risk of Bias

The risk of bias assessment for the selected studies was conducted using the Revised Cochrane Risk of Bias tool (RoB 2.0) for RCTs and the Risk of Bias in Non-randomized Studies of Interventions tool (ROBINS-I) for non-randomized studies [32]. Four researchers divided into two groups conducted the assessment, and any disagreements were resolved by re-examining the studies during weekly meetings. Funnel plots were used to evaluate the possibility of publication bias.

### 2.7. Data Synthesis and Statistical Analysis

For the systematic review, tables were used to organize and classify the study contents for the descriptive analysis. The meta-analysis was conducted using the R-4.1.1 program for Windows. In 13 studies, burnout was measured using the MBI, with 3 studies reporting total burnout scores, 11 measuring EE, 10 measuring DP, and 10 measuring PA. Six studies measured burnout using other methods. The meta-analysis applied both fixed- and random-effects models, reporting 95% confidence interval (CI), pooled standardized mean difference (SMD), and weights for each study. Heterogeneity across studies was calculated using the I^2^ index. A fixed-effects model was applied when heterogeneity was low (I^2^ < 50%), whereas a random-effects model was used when heterogeneity was moderate to high (I^2^ ≥ 50%). To account for variations in data formats across studies, separate meta-analyses were conducted for each burnout dimension (EE, DP, and PA) as well as for studies reporting total burnout scores. Sensitivity analyses were not conducted because the number of studies included for each outcome (ranging from 3 to 11) was insufficient to ensure stable pooled estimates. Subgroup analyses based on delivery format, intervention duration, or professional group were also considered but could not be performed for the same reason, as the number of studies within each category was too small to produce meaningful or reliable subgroup estimates This study received an exemption from review by the institutional review board (IRB) of the principal investigator’s institution.

### 2.8. Generative AI Disclosure

During the preparation of this manuscript, Generative AI was used to assist with language polishing and improving sentence clarity. The AI tool was not used for idea generation, interpretation of results, or reference creation. All content generated with AI assistance was carefully reviewed, edited, and verified by the authors. The authors take full responsibility for the accuracy and integrity of the manuscript.

## 3. Results

### 3.1. Study Characteristics

A total of 5005 studies were identified through the systematic literature search. After eliminating 1665 duplicates, titles and abstracts of 3340 publications were evaluated according to the inclusion and exclusion criteria. A total of 3233 studies were excluded for not meeting the selection criteria, and 107 relevant studies were selected based on the research objectives. After full-text review, 107 studies were excluded. Ultimately, 29 studies were selected; among them, 11 studies that measured at least one of the three burnout subdimensions—EE, DP, or PA—using the MBI were included in the meta-analysis.

Table 1 presents a comprehensive overview of the studies included in this systematic review. Of the 29 retrieved studies, 22 adopted an RCT design [13,27,33,34,35,36,37,38,39,40,41,42,43,44,45,46,47,48,49,50,51,52], whereas seven employed quasi-experimental designs [12,53,54,55,56,57,58]. Fourteen studies were published in North America [13,33,34,35,40,41,42,43,44,45,47,51,55,57], nine in Asia [37,38,39,49,50,52,54,56,58], four in Europe [27,46,48,53], and one each in Africa [12] and Australia [36]. Regarding participants, 16 studies recruited nurses [12,13,27,37,38,40,41,49,50,52,53,54,55,56,57,58], six physicians and residents [33,34,36,44,48,51], and seven interdisciplinary HPs [35,39,42,43,45,46,47]. The sample size ranged from 24 to 2182 participants for a total of 4545, with 2384 and 2161 in the experimental and control groups, respectively. Most participants were female within an age range of 22–60 years. The MBI was the most frequently used measure for burnout, with 23 out of 29 studies [12,13,27,33,34,35,37,38,40,41,42,43,44,46,47,48,49,50,51,52,56,57,58]. The control groups consisted of 14 studies with no intervention [12,33,34,35,37,41,43,45,47,51,54,56,57,58], eight studies with active control [36,38,39,46,49,50,52,55], and seven studies with wait-list control groups [13,27,40,42,44,48,53]. Among the studies with active controls, the comparison conditions varied and consisted of structured intervention programs, including an additional weekly break [36], psychoeducational or education-based sessions [38,50,52,55], digital or self-help programs such as psychological articles or the National Health Service (NHS) digital platform for work-related stress (Moodzone) [39,49], and a Nurse Resilience Program (NRP) delivered without the mindfulness component [46]. Most published studies were journal articles [12,13,27,33,34,35,36,37,38,39,40,41,42,43,44,45,46,47,48,49,50,51,52,53,54,55,56,58], and one study was a doctoral dissertation [57].

Table 2 presents the intervention characteristics of the included studies. Mindfulness-based interventions overwhelmingly predominated among the included studies, with 26 of the 29 studies implementing mindfulness as a standalone third-wave CBT [12,13,27,33,34,35,37,38,39,40,43,44,45,46,47,48,49,50,51,52,53,54,55,56,57,58], whereas three studies implemented a combination of mindfulness and other therapeutic modalities: CBT and ACT [36]; CBT [41]; and resilience training [42]. For the 29 mindfulness interventions, 22 provided interventions that were either newly developed in 14 studies [13,34,41,42,44,45,46,47,49,50,51,52,56,58], or adaptations of existing programs tailored to the participants in eight studies [35,36,38,39,40,54,55,57], whereas seven studies used existing mindfulness programs [12,27,33,37,43,48,53].

Sixteen studies delivered interventions on-site [34,35,36,38,40,41,43,45,48,50,51,52,53,54,56,58] whereas 12 studies utilized online delivery methods [12,13,27,33,37,39,42,46,47,49,55,57] and one study employed hybrid methods [44]. Among the 16 on-site interventions, 10 were conducted in a group format [34,35,36,38,40,43,48,52,53,54], whereas 6 studies were conducted in an individual format [41,45,50,51,56,58]. Twelve studies with online interventions were participant-directed. Among these, most were delivered asynchronously through self-paced online modules or mobile applications [12,13,27,33,39,42,46,47,55,57], while a few studies provided synchronous sessions such as live video conferences or real-time group meetings [37,49].

### 3.2. Intervention Characteristics

The duration of the interventions varied between 4 weeks and 6 months: nine studies lasted 4 weeks [13,38,39,40,49,54,55,56,57], four lasted 6 weeks [27,35,44,53], seven lasted 8 weeks [12,33,37,43,47,52,58], two lasted 10 weeks [36,45], four lasted 12 weeks [41,42,48,51], one lasted 13 weeks [50], one lasted 4.5 months [46], and one lasted 6 months [34].

The outcomes were measured immediately post-intervention in all 29 studies. Among these, 13 studies included follow-up assessments [12,27,34,39,45,46,49,50,51,52,53,54,56] with follow-up periods ranging from 1 month to 6 months or longer. Among the 16 studies delivered using the on-site method, 14 reported dropout rates [34,35,38,40,41,43,45,48,50,51,52,53,54,58], which ranged from 0% to 52.0%. Of the 12 studies delivered online [12,13,27,33,37,39,42,46,47,49,55,57], dropout rates ranged from 0% to 44.0%. In a study that delivered an intervention using a hybrid method [36], the dropout rate was 4.5%.

### 3.3. Risk of Bias

**Figure 2:**
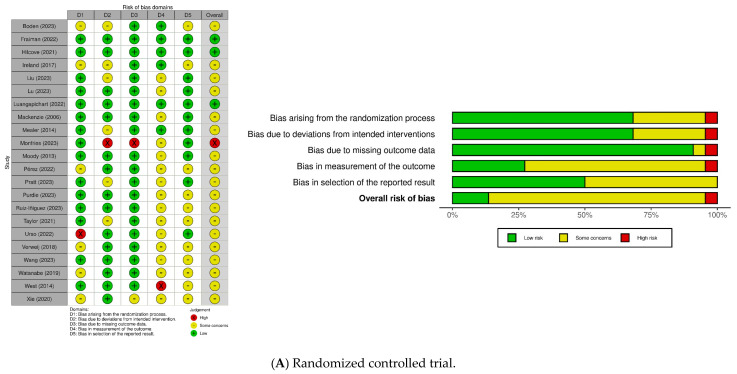

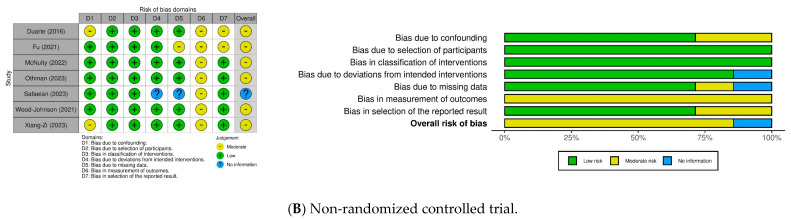
Quality appraisal. (generated using the robvis tool; https://mcguinlu.shinyapps.io/robvis/ (accessed on 30 October 2024)) (**A**): [13,27,33,34,35,36,37,38,39,40,41,42,43,44,45,46,47,48,49,50,51,52]; (**B**): [12,53,54,55,56,57,58].

**Figure 3:**
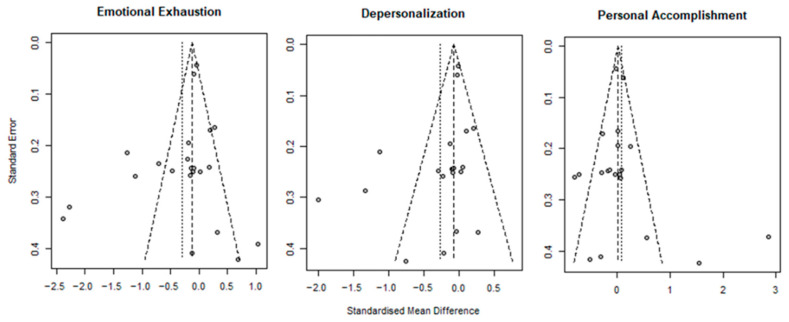
Funnel plots.

The risk of bias is shown in Table 1 and Figure 2. Among the 22 RCTs, the risk of bias was rated as “some concerns” in 17 studies [13,29,36,37,38,40,41,43,44,45,46,47,48,49,50,51,52], “low risk” in three studies [34,35,39], and “high risk” in two studies [27,42]. Of the seven quasi-experimental studies, six were rated as having a “moderate risk” of bias [12,53,54,55,57,58], whereas one study could not be assessed because of limited information [56]. Publication bias risk was evaluated using a funnel plot and Egger’s linear regression test (Figure 3). The funnel plot revealed a symmetric distribution of the studies around the pooled effect size, suggesting a low risk of publication bias. Although slight asymmetry was observed, this may be attributable to small study effects or heterogeneity among the included studies. Egger’s linear regression test indicated no statistically significant publication bias for EE, DP, or low PA (Table 3).

### 3.4. Meta-Analysis

**Figure 4:**
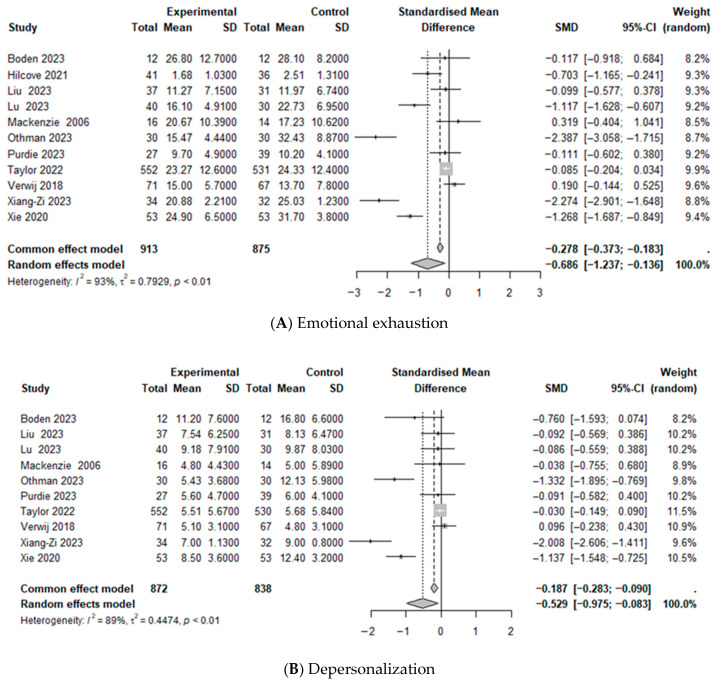
Forest plots: Effects of third-wave cognitive-behavioral therapy interventions on emotional exhaustion and depersonalization. (**A**): [12,33,35,37,38,40,44,46,48,52,58]; (**B**): [12,33,37,38,40,44,46,48,52,58].

This meta-analysis examined the effects of third-wave CBT on the following burnout dimensions: EE, DP, and low PA (Table 4, Figure 4). A meta-analysis of 11 studies that evaluated EE [12,33,35,37,38,40,44,46,48,52,58] and 10 studies that measured DP and low PA [12,33,37,38,40,44,46,48,52,58] revealed that the third-wave CBTs led to statistically significant reductions in EE (SMD = −0.686, CI = −1.237, −0.136, *p* < 0.05, I^2^ = 92.5%) and DP (SMD = −0.529, CI = −0.975, −0.083, *p* < 0.05, I^2^ = 89.3%). By contrast, the effect of low PA was not statistically significant.

## 4. Discussion

This study aimed to comprehensively evaluate the effectiveness of third-wave CBTs in reducing burnout among interdisciplinary HPs. This is the first meta-analysis to evaluate the effectiveness of third-wave CBTs on burnout among HPs.

Our meta-analysis results demonstrated significant reductions in EE and DP among HPs. Reductions in EE and DP appear clinically meaningful for healthcare professionals and may lead to better work functioning, lower turnover intention, and improved patient experience and safety. These findings are consistent with the effectiveness of third-wave CBTs for burnout, stress, depression, psychological distress, and job strain-related symptoms [24,59]. Reducing EE and DP may also contribute to improved workforce retention and lower absenteeism, ultimately benefiting healthcare providers and enhancing patient safety and care quality [9,10,11].

However, no significant effect of low PA was found in a meta-analysis of the retrieved studies. One possible explanation is that PA is a burnout dimension that is less responsive to psychological interventions such as CBT alone [60]. Unlike EE or DP, PA is more strongly influenced by structural and environmental factors, including work autonomy, recognition, opportunities for professional development, and organizational culture [60,61]. These external variables are often beyond the immediate scope of cognitive or behavioral therapy. Therefore, improving healthcare professionals’ sense of PA may require a dual approach that combines psychological support with systemic organizational change [62]. The Healthcare Labor Practice Framework also highlights that PA is closely tied to conditions such as workload, staffing adequacy, emotional labor demands, and autonomy [63]. These structural determinants limit the extent to which individual-level CBT can improve PA. Therefore, integrating third-wave CBT with organizational enhancements—such as supportive leadership, equitable workload distribution, and expanded opportunities for professional growth—may be necessary to produce meaningful improvements in PA.

Most third-wave CBTs sessions (26 out of 29 studies) included in this study were standalone mindfulness interventions, with only three studies combining mindfulness with other therapeutic interventions. This indicates that current research on third-wave CBTs relies heavily on one modality of intervention—mindfulness—with relatively few studies on other approaches, such as ACT or dialectical behavior therapy. This is understandable as most evidence of CBTs and burnout accumulates in mindfulness [62]. However, reliance on a single type of intervention limits our understanding of the broader therapeutic potential of third-wave CBTs. Future studies may benefit from examining other third-wave interventions and from exploring integrated interventions that combine multiple third-wave components where appropriate. In addition, combining psychological mechanisms (e.g., ACT-based psychological flexibility training) with organizational strategies—such as adjusted workload distribution, protected time for reflection, or supportive supervisory structures—may offer a more comprehensive way to address burnout, particularly the PA dimension, which is sensitive to workplace conditions.

The majority of the included studies utilized no intervention or wait-list groups as controls, with a relatively limited use of active control groups. This finding aligns with previous research demonstrating the effectiveness of mindfulness interventions in HPs [59,64]. However, the limited use of active control groups may compromise the rigor of comparisons as these groups better account for placebo effects and participant expectations [65]. Future trials should additionally incorporate longitudinal follow-up to determine whether improvements in EE and DP are sustained over time and to assess whether delayed improvements in PA emerge as organizational changes evolve within healthcare settings.

Despite differences in delivery modes, our results indicated no variation in dropout rates between on-site and online intervention studies. Consistent with our findings, previous studies investigating adherence to on-site versus online interventions reported no significant differences in dropout rates [66,67]. Given the growing use of third-wave CBT in workplace wellness programs, institutional support also plays a role. Providing protected time for participation and ensuring basic staffing coverage may help improve engagement and the overall feasibility of such interventions.

Overall, this review highlights that third-wave CBT interventions can meaningfully reduce EE and DP among healthcare professionals, whereas improvements in PA depend more heavily on organizational conditions. On the other hand, recent advances in machine-learning–based affective computing highlight an emerging methodological direction for burnout research. Recent studies have shown that transfer-learning approaches can substantially enhance facial-expression recognition performance, illustrating how AI-driven methods may support future advancements in detecting emotional strain and developing more tailored psychological interventions for healthcare professionals [68]. Future research should adopt integrated, multi-level approaches that combine psychological strategies with organizational enhancements to more effectively address all dimensions of burnout. Such efforts will support the development of practical, evidence-based programs that can be implemented within diverse healthcare settings.

### Strengths and Limitations

This study adopted a multidimensional approach by analyzing three burnout dimensions: EE, DP, and low PA. This analytical framework distinguishes itself from previous studies that have predominantly focused on aggregate burnout scores for meta-analyses [69]. By examining each dimension separately, this study provides a more sophisticated understanding of how third-wave CBTs impacts the specific burnout dimensions. Additionally, the inclusion of interdisciplinary HPs as participants allows for a comprehensive understanding of burnout across interdisciplinary healthcare teams rather than being limited to a single profession. These results can be used to design interventions tailored to reduce burnout.

Nevertheless, this study had several limitations. First, the high heterogeneity of interventions regarding duration, delivery methods, and measurement tools for outcomes among the included studies posed a challenge. Consistent with our pooled analysis, between-study heterogeneity was substantial, indicating that true effects likely vary across contexts. In six studies, interventions were conducted during the COVID-19 pandemic, which significantly influenced HPs’ workplaces. The intervention duration ranged from a minimum of 4 weeks to a maximum of 12 weeks. These differences were considered when interpreting our results. Furthermore, while most studies used the MBI to measure the burnout dimensions, some utilized alternative tools that contribute to variability in measuring outcomes. To better account for this variability, future research should consider prespecified subgroup analyses and meta-regression to explore potential moderators.

Second, the quality assessment of the included studies indicated potential bias. Using the RoB 2.0 and the ROBINS-I tools, some studies were rated as having issues and moderate risk, and one study could not be evaluated because of limited information due to inadequate blinding or allocation procedures. The ROBINS-I assessment identified potential confounding factors in nonrandomized studies, necessitating caution when interpreting the results. These limitations may affect the generalizability of our findings. Additionally, combining RCTs with quasi-experimental studies may introduce design-related bias, as the latter are more vulnerable to confounding and less rigorous in controlling for baseline differences. This methodological heterogeneity should be considered when interpreting the pooled findings.

Third, although subgroup or sensitivity analyses could have provided additional insight into sources of heterogeneity, these analyses were not feasible because the number of studies within each subgroup (e.g., by duration, delivery format, or professional group) was insufficient to produce meaningful or stable estimates. Consequently, the pooled effect sizes should be interpreted with caution, as the substantial between-study variability may influence the robustness of the results.

## 5. Conclusions

The meta-analysis results showed that third-wave CBTs significantly reduced EE and DP among HPs. Although our findings primarily reflect mindfulness-based interventions, which comprise the majority of the analyzed studies and can be delivered online through digital platforms, future research should explore the potential of other third-wave CBT approaches. This expansion of therapeutic approaches could provide HPs with more diverse and accessible interventions despite time constraints in clinical practice. 

Third-wave CBTs can be implemented through various delivery methods, including onsite, online, and hybrid formats. The growing prominence of digital health solutions, particularly since the onset of the COVID-19 pandemic, underscores the need for flexible and scalable interventions tailored to the specific needs and constraints of each healthcare institution. Accordingly, healthcare organizations should consider integrating evidence-based third-wave CBT programs into institutional well-being strategies to reduce burnout risk and enhance workforce sustainability. Developing standardized implementation protocols and validating delivery methods across settings will be essential to ensure both accessibility and effectiveness. However, further studies are required to explore the mechanisms underlying low PA and third-wave CBTs.

Our findings have several practical implications. For clinical practitioners, the implementation of third-wave CBTs should be considered when managing EE and DP among interdisciplinary HPs. Healthcare institutions should select appropriate delivery methods that align with their organizational characteristics and staff needs. RCTs comparing the effectiveness of different burnout dimensions and long-term follow-up studies are required to verify the sustainability of the intervention’s effects on burnout dimensions. Furthermore, the development of comprehensive intervention programs that focus on enhancing PAs is necessary.

## Figures and Tables

**Table 1:** Study characteristics.

No.	First Author (Year) Country	Study Design	Participants	Sample Size (Female %)	Age (Mean ± SD Years)	Controls	Third Wave CBT Type	Outcome Measures (Variables)	Publication Type	Risk of Bias
1	Boden (2023) United states [33]	RCT	Orthopedic surgery residents	24 (E = 12, C = 12) (37.5%)	E: 31.0 ± 2.8 C: 30.6 ± 2.8	No intervention	Mindfulness	MBI (burnout), PSS (stress), GAD-7 (anxiety)	Journal article	Some concerns
2	Duarte (2016) Portugal [53]	Quasi experimental	Oncology nurses	48 (E = 29, C = 19) (90.1%)	E: 38.90 ± 8.34 C: 42.11 ± 8.43	Wait-list	Mindfulness	ProQOL-5 (burnout, compassion fatigue, secondary traumatic stress), DASS-21 (depression, anxiety, stress), AAQ-II (acceptance), RRS (ruminative responses), FFMQ (mindfulness), SCS (self-compassion), SWL (satisfaction with life)	Journal article	Moderate
3	Fraiman (2022) United States [34]	RCT	Pediatric resident	340 (E = 194, C = 146) (75%)	Mean age not provided; 15% were 30 years or older	No intervention	Mindfulness	-Primary: MBI (emotional exhaustion) -Secondary: MBI (depersonalization, personal accomplishment), FFMQ (mindfulness), IRI (empathy)	Journal article	Low
4	Fu (2021) Taiwan [54]	Quasi experimental	Registered nurses	124 (E = 67, C = 57) (No percentage of females)	E: 39.01 ± 9.27 C: 37.09 ± 8.53	No intervention	Mindfulness	ProQOL-5 (burnout, compassion fatigue, secondary traumatic stress), SF-12v2 (mental health, physical health)	Journal article	Moderate
5	Hilcove (2021) United States [35]	RCT	Nurses and healthcare professionals	78 (E = 41, C = 37) (95%)	Both groups: 42.41 ± 12.12 (range: 24–69)	No intervention	Mindfulness	MBI (burnout), PSS (stress), SF-36 (vitality), GSQI (sleep quality), BSS (serenity), MAS (mindfulness), Biomarkers (diurnal salivary cortisol, Blood pressure)	Journal article	Low
6	Ireland (2017) Australia [36]	RCT	Intern doctors	44 (E = 23, C = 21) (64%)	26.88 ± 4.79 (range: 22–48)	Active control (weekly 1 h break)	MBSR, MBCT, ACT	CBI (burnout), PSS (stress),	Journal article	Some concerns
7	Liu (2023) China [37]	RCT	Registered nurses	68 (E = 31, C = 37) (98.5%)	38.11 ± 7.81 (range: 24–59)	No intervention	Mindfulness	MBI (burnout), SWB scale, Psychological adaptability questionnaire	Journal article	Some concerns
8	Lu (2023) China [38]	RCT	ICU nurses	70 (E = 40, C = 30) (92.2%)	E: 28.33 ± 3.32 C: 27.27 ± 3.76	Active control (Psycho-educational)	Mindfulness	MBI-HSS (burnout), FFMQ (mindfulness), SAS (anxiety), CESD (depression), PANAS, SWLS (subjective well-being)	Journal article	Some concerns
9	Luangapichart (2022) Thailand [39]	RCT	Medical personnel	90 (E = 45, C = 45) (84.4%)	E: 34.07 ± 8.49 (Group A) C: 32.87 ± 7.27 (Group B)	Active control (Group B -Psychological self-help articles)	Mindfulness	CBI (burnout), ST-5 (stress), HADS (anxiety, depression), PHLMS (mindfulness), WHOQOL-BREF (quality of life)	Journal article	Low
10	Mackenzie (2006) Canada [40]	RCT	Nurses and nurse aides	30 (E = 16, C = 14) (97%)	E: 48.62 ± 6.52 C: 44.78 ± 8.16	Wait-list	Mindfulness	MBI (burnout), SRDI (relaxation), JSS (job satisfaction), SWLS (life satisfaction), OLQ (sense of coherence)	Journal article	Some concerns
11	McNulty (2022) United States [55]	Quasi experimental	Newly graduated nurses	200 (E = 131, C = 69) (E = 82.4%, C = 86.9%)	E: 27.16 ± 6.79 C: 26.2 ± 6.55	Active control (NRP without mindfulness)	Mindfulness	OBI, PWLSSI (burnout), PSS (stress), MAAS (mindfulness), turnover rate, CFGNES (overwhelm and stress)	Journal article	Moderate
12	Mealer (2014) United States [41]	RCT	ICU nurses	27 (E = 13, C = 14) (E = 92%, C = 86%)	N/A	No intervention	CBT, MBSR	MBI (burnout), CD-RISC (Resilience), HADS (anxiety, depression), PDS (PTSD symptoms)	Journal article	Some concerns
13	Monfries, (2023) Canada [42]	RCT	Healthcare professionals	34 (E = 17, C = 17) (81.6%	N/A	Wait-list	Mindfulness and resilience training	MBI (burnout), CDRS (resilience), MAAS (mindfulness)	Journal article	High
14	Moody (2013) United States [43]	RCT	Pediatric oncology clinical staff	45 (E = 21, C = 24) (80%)	N/A	No intervention	Mindfulness	-Primary: MBI (emotional exhaustion) -Secondary: BDI (depression), PSS (stress), MAAS (mindfulness)	Journal article	Some concerns
15	Othman (2023) Egypt [12]	Quasi experimental	Critical care nurses	60 (E = 30, C = 30) (75%)	N/A	No intervention	Mindfulness	MBI (burnout), FFMQ (mindfulness), SCS (self-compassion)	Journal article	Moderate
16	Pérez (2022) Spain [27]	RCT	Nurses	74 (E = 39, C = 35) (89.6%)	37 ± 9.13	Wait-list control group	Mindfulness	MBI (burnout), PQLS (quality of life)	Journal article	High
17	Pratt (2023) United States [13]	RCT	Nurses	102 (E = 69, C = 33) (94%)	26.5 (range: 24–32)	Wait-list	Mindfulness	MBI (burnout), PHQ-9 (depression), PSS (stress), Feasibility	Journal article	Some concerns
18	Purdie (2023) United States [44]	RCT	Pediatric resident physicians.	66 (E = 27, C = 39) (78.8%)	E: 26 C: 37	Wait-list	Mindfulness	-Primary: PSS (stress) -Secondary: MBI (burnout), BDI (depression), UCLA Loneliness Scale, PSQI (sleep quality).	Journal article	Some concerns
19	Ruiz-Iñiguez (2023) Cuba [45]	RCT	Mental health professionals	104 (E = 52, C = 52) (90.3%)	41 ± 11.91	No intervention	Mindfulness	BBQ (burnout), SSS (stress), STAI-S (anxiety)	Journal article	Some concerns
20	Safaeian (2023) Iran [56]	Quasi experimental	Female nurses	60 (E = 40, C = 20) (100%)	28.95 ± 3.18	No intervention	Mindfulness	Cognitive fusion, MBI (burnout)	Journal article	No information
21	Taylor (2022) United Kingdom [46]	RCT	Employees within an NHS trust or GP practice	2182 (E = 1095, C = 1087) (83%)	40.42 ± 10.92	Active control (The NHS digital platform for work-related stress, Mood-zone)	Mindfulness	DASS-21 (depression, anxiety, stress), SWEMWBS (Mental well-being), MBI (burnout), FFMQ-15 (mindfulness), SCS-SF (Self-compassion), CLS (compassionate love), PSWQ (worry), RRS (brooding), Sickness absence	Journal article	Some concerns
22	Urso (2022) United states [47]	RCT	Nurses and patient care technicians	45 (E = 24, C = 21) (78%)	N/A	No intervention	Mindfulness	DASS-21 (depression, anxiety, stress), MBI-HSS (burnout)	Journal article	Some concerns
23	Verweij (2018) Netherlands [48]	RCT	Residents from all medical, surgical, and primary care disciplines	148 (E = 80, C = 68) (88%)	31.2 ± 4.6	Wait-list	Mindfulness	-Primary: MBI-HSS, UBOS-C (burnout) -Secondary: PSWQ (worry), FFMQ (mindfulness), SCS (self-compassion), mental health continuum, JSPE (empathy)	Journal article	Some concerns
24	Wang (2024) China [49]	RCT	Psychiatric nurses	118 (E = 59, C = 59) (88%)	32.25 ± 3.56	Active control (Psychoeducational)	Mindfulness	MBI-HSS (job burnout), FFMQ (mindfulness), CD-RISC (resilience)	Journal article	Some concerns
25	Watanabe (2019) Japan [50]	RCT	Junior nurses	80 (E = 40, C = 40) (100%)	30.1 ± 8.4	Active control (Psychoeducation group)	Mindfulness	MBI-HSS (burnout), GAD-7 (anxiety), HADS (anxiety, depression), ISI (insomnia), PHQ-9 (depression)	Journal article	Some concerns
26	West (2014) United States [51]	RCT	Practicing physicians	74 (E = 37, C = 37) (33.9%)	N/A	No intervention	Mindfulness	Engagement at work, MBI (burnout), PSS (stress), depression, QOL fatigue, PJSS (job satisfaction), JSPE (empathy), Medical Outcomes Study Short-Form Health Survey	Journal article	Some concerns
27	Wood-Johnson (2021) United States [57]	Quasi experimental	Registered nurses (RNs)	38 (E = 25, C = 13) (No percentage of females)	24 to 60 years (No mean age)	No intervention	Mindfulness	MBI (burnout)	Doctoral dissertation	Moderate
28	Xiang-Zi (2023) China [58]	Quasi experimental	Intensive care unit nurses	66 (E = 34, C = 32) (72.7%)	29.72 ± 1.81	No intervention	Mindfulness	Death anxiety, MBI-HSS (burnout)	Journal article	Moderate
29	Xie (2020) China [52]	RCT	Intensive care unit nurses	106 (E = 53, C = 53) (100%)	27.7 ± 7.7	Active control (Education-based group)	Mindfulness	MBI (burnout), MAAS (mindfulness), ACQ (acceptance, action)	Journal article	Some concerns

Notes: AAQ-II, acceptance and action questionnaire–II; ACQ, acceptance and action questionnaire-II; ACT, acceptance and commitment therapy; BBQ, brief burnout questionnaire; BDI, beck depression inventory; CBI, Copenhagen-burnout inventory; CBT, cognitive behavioral therapy; CDRS, Connor-Davidson resilience scale; CESD, center for epidemiological studies depression scale; CFGNES, Casey-Fink graduate nurse experience survey; CLS, compassionate love scale; DASS-21, depression, anxiety, stress scale; FFMQ, five facets of mindfulness questionnaire; GAD-7, generalized anxiety disorder-7; GSQI, global sleep quality item; HADS, hospital anxiety and depression scale; ICU, intensive care unit; IRI, interpersonal reactivity index; ISI, insomnia severity index; JSS, intrinsic job satisfaction subscale of the job satisfaction scale; JSPE, Jefferson scale of physician empathy; MAAS, mindful attention awareness scale; MBCT, mindfulness-based cognitive therapy; MBI, Maslach burnout inventory; MBI-HSS, Maslach burnout inventory-human services survey; MBSR, mindfulness-based stress reduction; MAS, mindfulness awareness survey; NRP, nurse residency program; OLQ, orientation to life questionnaire; OBI, Oldenburg burnout inventory; PANAS, positive affect and negative affect scale; PDS, posttraumatic diagnostic scale; PHLMS, Philadelphia mindfulness scale; PHQ-9, patient health questionnaire-9; PJSS, physician job satisfaction scale; PQLS, professional quality of life scale; ProQOL-5, professional quality of life scale-5; PSQI, Pittsburgh sleep quality index; PSS, perceived stress scale; PSWQ, Penn state worry questionnaire; PWLSSI, physician work-life study single item; QOL, quality of life; RCT, randomized controlled trial; RRS, ruminative responses scale-short; SAS, self-rating anxiety scale; SCS, self-compassion scale; SF-12v2, short-form 12 item version 2; SF-36, vitality subscale of the medical outcomes study short form-36; SRDI, smith relaxation dispositions inventory; SSS, symptomatic stress scale; ST-5, stress test questionnaire; STAI-S, anxiety state inventory; SWB, satisfaction with life scale; SWEMWBS, short Warwick Edinburgh mental well-being scale; SWLS, satisfaction with life scale; UBOS-C, Utrecht burnout scale.

**Table 2:** Intervention characteristics.

No.	First Author (Year)	Source of Intervention	Mode of Delivery	Intervention Period and Intervention Dose	Follow-Up Time Points	Dropout Rate%	Impact of COVID-19
1	Boden (2023) [33]	Existing mindfulness programs—Mindfulness based application “Headspace”	Online, through a mobile app (audio)	-Period unspecified -Daily for 2 months (On average, 7.9 ± 6.6 min per day, 2 days per week)	Baseline and post-intervention (after 2 months)	0%	Not mentioned
2	Duarte (2016) [53]	Existing mindfulness programs—MBSR by Jon Kabat-Zinn	On-site, group sessions, with supplementary CD for home practice (audio)	-Between 2013 and 2015 -6 weeks (Six 2 h group sessions), with daily home practice of at least 15 min	Pre, post intervention (week 6), and 3-month follow-up	52%	Not applicable (study conducted before the pandemic)
3	Fraiman (2022) [34]	Developed Kern’s six-step approach	On-site, group sessions during existing didactic time	-14 June 2017 to 28 February 2019 -6 months (7 h-long sessions), plus optional mindfulness refreshers	Baseline, post-intervention (month 6), and follow-up (month 15)	-19.7% at 6 months -42.6% at 15 months	Not applicable (study conducted before the pandemic)
4	Fu (2021) [54]	Adaptations of existing programs -Mindfulness respiration (Kabat-Zinn) -Compassion fatigue resiliency (Gentry et al.)	On-site, group sessions with supplementary materials for home practice	-May 2017 to December 2017 -4 weeks (4 sessions 2 h each), plus additional self-practice encouraged	Baseline, end of intervention (4 weeks), 4 weeks post-intervention, and 12 weeks post-intervention	-E: 3% at the end of intervention, 24% at 4 weeks, 42% at 12 weeks -C: 0% at the end of intervention, 11% at 4 weeks, 39% at 12 weeks)	Not applicable (study conducted before the pandemic)
5	Hilcove (2021) [35]	Adaptations of existing programs -Incorporating elements of Hatha and Raja Yoga, based on principles outlined by Jon Kabat-Zinn	On-site, group sessions with supplementary DVD/CD for home practice	-Period unspecified -6 weeks (weekly sessions, approximately 137 min per week of home practice on average)	Baseline and post-intervention (6 weeks)	2.5%	Not mentioned
6	Ireland (2017) [36]	Adapted from well-validated psychological treatment programs for a non-clinical population	On-site, group sessions with additional self-directed practice recommended	-Period unspecified -10 weeks (weekly sessions, with encouragement to practice mindfulness outside of sessions)	Baseline, mid-intervention (week 5), and post-intervention (week 10)	N/A	Not applicable (study conducted before the pandemic)
7	Liu (2023) [37]	Existing mindfulness programs–MBSR by Jon Kabat-Zinn	Online, Internet and WeChat-an app that allows social networking through text, voice, and video	-July 2022 -8 weeks (Each session lasted 1.5 to 2 h)	Baseline and post-intervention (8 weeks)	24.44%	Not mentioned
8	Lu (2023) [38]	Adapted from the standard MBSR program developed by Jon Kabat-Zinn, combined with loving-kindness meditation	On-site group sessions, with participants encouraged to practice mindfulness at home using provided audio resources	-Between 2016 and 2017 -4 weeks (8 sessions, 2 h each), with daily home practice encouraged (at least 20 min per day)	Baseline (T1), post (T2), 2 months post (T3), and 6 months post intervention (T4)	22.2%	Not applicable (study conducted before the pandemic)
9	Luangapichart (2022) [39]	Adaptations of existing programs -Incorporating Dynamic Meditation principles by Luangpor Teean Jittasubho	Online, daily guided mindfulness practices via LINE app	-June 2021 to October 2021 -4 weeks (Daily mindfulness practice for 28 days), with each audio session to be repeated at least three times a day	Group A—Baseline (T0), 4 weeks (T1), 8 weeks (T2); Group B-Baseline (T0), 4 weeks (T1), 8 weeks (T2), 12 weeks (T3), and 16 weeks (T4)	4.4%	The study was conducted during the pandemic, which influenced the recruitment and stress levels of participants.
10	Mackenzie (2006) [40]	Adapted from traditional MBSR programs as developed by Jon Kabat-Zinn	On-site, group sessions with supplementary materials for home practice	-Period unspecified -4 sessions (30 min each), with recommended daily home practice	Baseline and post-intervention (4 weeks)	0%	Not applicable (study conducted before the pandemic)
11	McNulty (2022) [55]	Adaptations of existing programs -From MBSR and related programs, designed specifically for NGNs	Online, virtual delivery via a secure online platform with supplementary email reminders for home practice	-Between April and June 2020 -4 weekly sessions (1 h each) with ongoing daily mindfulness practice encouraged	Baseline (pre-intervention) and 6 months post-intervention	0%	The COVID-19 pandemic shifted the study to a virtual format and increased participants’ stress levels.
12	Mealer (2014) [41]	Developed by the authors, incorporating CBT, MBSR, expressive writing, and exercise	On-site, combined with at-home practices	-October 2012 to December 2012 -12 weeks, including a 2-day workshop	Baseline and post-intervention (12 weeks) (No long-term follow-up)	0%	Not applicable (study conducted before the pandemic)
13	Monfries, (2023) [42]	Developed modules on mindfulness, self-expertise, mental fitness, mental health, hardiness, and energy management	Online through a smartphone app with self-directed participation (via the Headversity™ App)	-March to June 2021 -12 weeks of self-paced engagement with the app’s content	Baseline and post-intervention (12 weeks) (No long-term follow-up)	44%	N/A
14	Moody (2013) [43]	Existing mindfulness programs—MBSR by Jon Kabat-Zinn	On-site, in-person group sessions at the hospital	-Not explicitly stated, but the study began in December 2011 -Total of 15 h of class time (one 6 h session, 6weekly 1 h follow-up sessions, and a final 3 h wrap-up session)	Measurements were taken at baseline and after the 8-week course; qualitative analysis of diaries provided additional insights	2%	N/A
15	Othman (2023) [12]	Existing mindfulness programs—MBSR by Jon Kabat-Zinn	Online delivery via WhatsApp	-2 month (8 weeks) -April to June 2021 -2.5 h per session	Baseline and post-intervention, follow up at 8 weeks	15.5%	During COVID-19, designed to address related stress. Available online
16	Pérez (2022) [27]	Existing mindfulness programs—MBSR by Jon Kabat-Zinn	Online, using videos, audio downloads, and emails.	-6 weeks -6 session (60 min per session)	Measurements at baseline, six weeks, and three months post-intervention	0%	N/A
17	Pratt (2023) [13]	Developed—LIFT: A mindfulness program to reduce symptoms of psychological distress and improve quality of life for intensive care unit (ICU) survivors	Online, through a mobile app with audio and video content	Access to Lift content after randomization for one month (4 weeks)	Baseline and one-month post-enrollment.	13.7%	The study was conducted during the COVID-19 pandemic but does not mention specific changes due to COVID-19
18	Purdie (2023) [44]	Developed—Mindful Awareness Practices (MAPs) developed at UCLA’s Mindful Awareness Research Center	Hybrid, on-site and digital (via secure mobile app)	-6 weeks -6 session (60 min per session)	Baseline and post-intervention	4.5%	N/A
19	Ruiz-Iñiguez (2023) [45]	Developed by the authors, guided by works of Kabat-Zinn and Segal	On-site sessions with audio guides for home practice	-January to March 2018 -10 weeks (2.5 h per session) -Plus home practice	Baseline, Posttest1 (week 6), Posttest2 (week 11), 6-month follow-up (week 37–39)	28.8%	N/A
20	Safaeian (2023) [56]	Developed based on the Kabat-Zinn and Young et al. for schema therapy	On-site sessions	-Conducted in 2021 -4 weeks -8 Sessions -1 h per session	Baseline, post-test, and 2-month follow-up	N/A	N/A
21	Taylor (2022) [46]	Developed—Unguided digital mindfulness-based self-help (MBSH) interventions	Online, website and mobile apps	Participants were encouraged to engage in at least one ten-minute mindfulness practice daily for the study duration (4.5 month)	Baseline (Time 1), after the initial intervention period (1.5 months, Time 2), and at post-intervention (4.5 months, Time 3).	35.1%	N/A
22	Urso (2022) [47]	Developed—LOTUS (Living Optimally in Times Under Stress) was developed by a yoga therapist and a yoga instructor	Online	-30 September 2019–21 January 2020 -Classes were on-site, 1 h duration with a cadence of once per week for a total of 8 weeks	Baseline and Post-intervention	6.6%	Due to the COVID-19 pandemic, the second group was not able to receive the MBI
23	Verweij (2018) [48]	Existing mindfulness programs–MBSR by Jon Kabat-Zinn	On-site, in-person group sessions	Daily practice at home for 45 min	baseline, post-intervention (approximately 3 months later)	15%	N/A
24	Wang (2024) [49]	Developed based on mindfulness-based cognitive interventions and mindfulness-based stress reduction programs	Online through WeChat, including audio and text materials.	-January to August 2022 -at home 5 days -8 weeks.	baseline, 4 weeks, and 8 weeks	16%	N/A
25	Watanabe (2019) [50]	Bishop’s mindfulness attention regulation combined with Beck’s CBT model.	On-site sessions conducted by senior nurses.	-13 weeks -4 sessions, -30 min per session	Baseline 13 weeks 26 weeks 52 weeks	5%	N/A
26	West (2014) [51]	Developed by the study authors, incorporating mindfulness, reflection, shared experience, and small-group learning	On-site sessions facilitated by trained internal medicine physicians.	-September 2010 and June 2012 -1 h biweekly sessions, totaling 19 sessions over 9 months	-Baseline—every 3 months through the 9-month study intervention -3 and 12 months post-study.	2.7%	N/A
27	Wood-Johnson (2021) [57]	Adaptations of existing programs -Palouse’s MBSR Program	Online through pre-recorded videos.	-22 February 2021, to 2 April 2021 -6 weeks -three times a week -Varying in length from 2 min to 60 min	-Baseline -3 weeks after intervention	18.4%	The study was conducted during the COVID-19 pandemic
28	Xiang-Zi (2023) [58]	Developed specifically for the study, based on existing mindfulness practices	On-site sessions conducted by a qualified psychologist.	-During the nurses’ work hours -45–50 min per session	baseline and post-intervention (2 months after baseline)	0%	N/A
29	Xie (2020) [52]	Developed-Based on MBSR, MBCT, ACT, and loving-kindness and compassion meditation	On-site group sessions	-October 2017 to May 2018 -8 weeks	-Baseline -Post-intervention -1 month after intervention -3 months after intervention	14.15%	N/A

Notes: ACT, acceptance and commitment therapy; C, control group; CBT, cognitive behavioral therapy; E, experimental group; MAP, mindful awareness practices; MBCT, mindfulness-based cognitive therapy; MBI, Maslach burnout inventory; MBSH, mindfulness-based self-help; MBSR, mindfulness-based stress reduction; N/A, not available.

**Table 3:** Estimated results of the Eggers linear test.

Burnout	t	df	*p*-Value
Emotional exhaustion	−1.46	20	0.1594
Depersonalization	−2.01	18	0.0597
Personal accomplishment	0.37	18	0.7171

**Table 4:** Combined SMDs and heterogeneity test in common effect model and random effects model.

Burnout	N	Common Effects Estimate		Heterogeneity			Random Effects Estimate	
		SMD (CI)	*p*-Value	Q	I^2^(%)	*p*-Value	SMD (CI)	*p*-Value
EE	11	−0.278 (−0.373, −0.183)	<0.0001	133.42	92.5	<0.0001	−0.686 (−1.237, −0.136)	0.0145
DP	10	−0.187 (−0.283, −0.090)	0.0001	83.95	89.3	<0.0001	−0.529 (−0.975, −0.083)	0.0202
PA	10	0.113 (0.016, 0.209)	0.0221	85.29	89.4	<0.0001	0.311 (−0.319, 0.941)	0.3338

Notes: EE = Emotional Exhaustion; CI = Confidence Intervals; DP = Depersonalization; PA = Personal accomplishment; SMD = Standardized Mean Difference.

## Data Availability

No new data were created or analyzed in this study. Data sharing is not applicable to this article as all data were obtained from previously published studies available in public databases.

## Abbreviations

The following abbreviations are used in this manuscript:

ACT	Acceptance and commitment
CBTs	Cognitive behavioral therapies
DP	Depersonalization
EE	Emotional exhaustion
HPs	Healthcare professionals
MBI	Maslach Burnout Inventory
PA	Personal accomplishment
RCT	Randomized controlled trial
